# A Multifunctional Adaptive and Interactive AI system to support people living with stroke, acquired brain or spinal cord injuries: A study protocol

**DOI:** 10.1371/journal.pone.0266702

**Published:** 2022-04-11

**Authors:** Giovanni Ottoboni, Fabio La Porta, Roberto Piperno, Rabih Chattat, Annalisa Bosco, Patrizia Fattori, Alessia Tessari

**Affiliations:** 1 Department of Psychology “Renzo Canestrari”, University of Bologna, Bologna, Italy; 2 IRCCS Istituto delle Scienze Neurologiche di Bologna, UO di Medicina Riabilitativa e Neuroriabilitazione, Bologna, Italy; 3 Department of Biomedical and Neuromotor Sciences, University of Bologna, Bologna, Italy; University of Ottawa, CANADA

## Abstract

**Background:**

Acquired brain injury and spinal cord injury are leading causes of severe motor disabilities impacting a person’s autonomy and social life. Enhancing neurological recovery driven by neurogenesis and neuronal plasticity could represent future solutions; however, at present, recovery of activities employing assistive technologies integrating artificial intelligence is worthy of examining. MAIA (*Multifunctional*, *adaptive*, *and interactive AI system for Acting in multiple context*s) is a human-centered AI aiming to allow end-users to control assistive devices naturally and efficiently by using continuous bidirectional exchanges among multiple sensorimotor information.

**Methods:**

Aimed at exploring the acceptability of MAIA, semi-structured interviews (both individual interviews and focus groups) are used to prompt possible end-users (both patients and caregivers) to express their opinions about expected functionalities, outfits, and the services that MAIA should embed, once developed, to fit end-users needs.

**Discussion:**

End-user indications are expected to interest MAIA technical, health-related, and setting components. Moreover, psycho-social issues are expected to align with the technology acceptance model. In particular, they are likely to involve intrinsic motivational and extrinsic social aspects, aspects concerning the usefulness of the MAIA system, and the related ease to use. At last, we expect individual factors to impact MAIA: gender, fragility levels, psychological aspects involved in the mental representation of body image, personal endurance, and tolerance toward AT-related burden might be the aspects end-users rise in evaluating the MAIA project.

## Introduction

According to the World Report on Disability of the World Health Organization, the global prevalence of disability is almost 20% [1]. In western societies, the most frequent causes of severe motor disabilities with the loss of independence are acquired brain injuries (vascular or traumatic) and spinal cord injuries (both traumatic and non-traumatic; hereafter SCI) [2]. The estimated incidence of traumatic brain injuries (TBI), stroke, and SCI in 2016–2017 were respectively 315, 162, and 27 new patients for 100 thousand people per year [3,4]. At a European level, 6.6% of the population older than 15 years has a severe motor disability, defined as a severe limitation or inability to walk and/or climb stairs. The prevalence of disability increases significantly with age: more than one-third of people dependent on mobility and personal care are older than 75 years, half of them are women (43.7%) [5]. Severe mobility limitations drive significant restrictions to social life. Less than half of people with severe limitations can count on supportive relational networks. Since many people with disabilities (PWD) live alone (27.4%), living with family members or other persons cannot secure sufficient functional levels of personal autonomy. Despite the alarming data on disability and the progress of knowledge related to neurogenesis and neuronal plasticity, no short-term solutions can firm a complete recovery based on the regeneration of damaged nerve tissue [6]. Therefore, in the non-acute phase of disabling diseases, enhancing functional activities could better fit the participation goals rather than the neurological recovery of impairments.

In this scenario, Assistive technologies (AT) represent gainful solutions. AT cluster all those objects, equipment, or systems that can be used to augment, stabilize or increase the functional capacities of a person with a disability [7]. Alternatively, AT are "*All equipment or devices used by an individual to help with the completion of an activity because of a health condition*" [8].

While hoping for tissue regeneration technologies to become viable solutions soon, AT systems composed of AI protocols mounting "intelligent neuroprostheses" are attracting research attention at present times [9,10]. Intelligent neuroprostheses are robotic devices forged with AI protocols to restore lost sensorimotor functions by acting for the damaged neuronal networks. In particular, these devices can capitalize on signals recorded from the brain and translate them into movements of electronic arms, legs or wheels, and tables [11,12]. From the early 2000s, brain-machine interfaces fostered neural signals’ acquisition to plan and execute motor actions. The primary records occurred from the frontal cortex [11,13–15]. However, their success in gesture planning did not result in gesture execution. The decoding from neurons obtained from the Posterior Parietal Cortex (PPC) seems more successful [16,17]: the sensory and motor information effectively guided a robotic arm moved by a person with tetraplegia [18,19].

The studies investigating the plans to match end users’ needs and AT have shown some limits over the years. Regarding the AT on the market, the drop-off rate affecting technologies was high if the person-and-technology matching was not finely tuned [7]. Some obstacles hampering AT adoption are poor control of AT cost, end users’ physicality, product security, and reliability. In particular, research suggests focusing on analyzing end users’ attitudes towards AT, the experience of control, the ease at use, the matching between the needs featuring both end-users and their primary caregivers, and solutions AT can offer [7]. Moreover, independence and autonomy emerged to represent the primary factors end-users expect AT to secure [20]. The evidence about the psycho-social plans to ensure the acceptability of intelligent neuroprostheses is increasing [21–24]. The advent of these systems has offered the possibility to control external devices (i.e. robotic arm) by using the patient’s brain activity [25]. Several studies showed the use of cortical signals extracted by motor brain areas in humans to reconstruct movement trajectories and endpoint goals [15,26–28]. However, only a few studies in humans focused on the possibility to extract neural signals from PPC to decode action intentions and action goals [18,27].

The present protocol is part of a HORIZON 2020 funded project situated along this vein (***M****ultifunctional* ***A****daptive and* ***I****nteractive* ***A****I system for acting in multiple context*s–MAIA—grant n. 951910). The project aims to develop a human-centric artificial intelligence (AI) to control prosthetic and assistive devices fitting on robotic arms, wheelchairs, and exoskeletons and help people with severe motor disabilities accomplish functional tasks. MAIA AI technology will decode human intentions and communicate the decoded targets to assistive devices and users to ensure compliance and develop trust through natural interaction and mutual learning [29,30]. In particular, it will combine sensorimotor inputs recorded from PPC with data regarding eye movements to provide end-users with the control of robotic devices. Furthermore, such human-centered AI will interact and have continuous bidirectional exchanges using gaze and other appropriate bahevioural parameters for action selection in the AI system, natural communication between a user and AI system and mutual learning [31]. These features ensure that the AI naturally and efficiently controls the neuroprosthesis or the assistive device motor output according to the end user’s intention and real needs. MAIA AI technology will decode human intentions and communicate the decoded targets to assistive devices and users to ensure compliance and develop trust through natural interaction and mutual learning.

As many AT shows, even MAIA might present problems in the adoption plans. For example, one of the main barriers impacting the adoption of AT concerns the degree of adherence between technological offers and end users’ needs [7], especially when the primary need is regaining autonomy and participation in social life [20]. Aside from this, cost-related issues, technology accessibility, and offer appropriateness need to be accounted for [32]. In this light, AT capable of responding to the end user’s needs must be developed upon a thorough analysis of their needs: Adopting a user-centered design method is critical in the project [32–35]. Therefore, to create a trustworthy human-centric AI technology, it is essential to acquire information from potential end-users, either patients or caregivers, on their attitudes towards AT and AI-driven systems, their expectations, and suggestions for future development. Thus, MAIA’s approach will be guided by the analysis of both end-users’ needs and device-related expectations through the direct involvement in the research program.

## Materials and methods

As the project aims to provide a reliable multifunctional AI controller for technological products such as robotic arms, electronic wheelchairs, and exoskeletons, the present protocol collects qualitative data about end users’ opinions. In particular, the latter may regard expected functionalities, outfits, and services that the AT should embed, once developed, to respond to end users’ needs successfully. The project protocol capitalizes on semi-structured interviews with PWD and primary caregivers: They can be met singularly or gathered in focus groups. Participants are interviewed through online software.

### Participants

The participants’ group involves PWD and primary caregivers. Among the PWD, people with TBI, stroke, or SCI are invited to participate. Participants are selected according to inclusion and exclusion criteria. No control group is involved in the study. The recruiting of the caregiver may be additional or separate from the PWD.

The PWD, enrolled by the Neurorehabilitation Unit of the Institute for Neurological Sciences of Bologna (’Istituto delle Scienze Neurologiche di Bologna’, ISNB) are cared for within coordinated multidisciplinary Care Pathways active in the metropolitan area of Bologna and specifically dedicated to "Severe brain injury", "Spinal Cord injury" and "Stroke". Patients and their caregivers are recruited if they meet all inclusion criteria and are keen to participate in the study. Furthermore, the potential participants are informed about participation in the study during the check-up visits scheduled by ISNB medical doctors referring the patients. Moreover, participants can also receive study advertisements from local associations gathering people with TBI, stroke, and SCI.

### Inclusion criteria

PWD must be 18 to 80 years old and suffer from acquired disability due to traumatic, non-traumatic SCI, ischemic or hemorrhagic stroke, or severe brain injury (traumatic and non-traumatic). In addition, the disability must have occurred six months to ten years before and be moderate to severe, as established by a score ranging from 3 to 5 on the Modified Rankin Scale. Finally, PWD must understand and sign the written consent for enrolment.

PWD’s caregivers must be aged from 18 to 80 years old, be the caregivers of PWD that match the previous criteria and have signed the written consent.

### Exclusion criteria

PWD presenting at least one of the following exclusion criteria are not be enrolled in the study:

- Severe psychiatric (e.g., psychosis, depression, apathy) and behavioral disorders (i.e., severe psychomotor agitation), cognitive disorders, or a state of confusion defined by temporal and/or spatial disorientation detected during an ordinary conversation. A simple confusion state assessment test (4AT) is administered in case of doubt [36].
- Language comprehension skills below 75% in an ordinary conversation due to aphasic disorder or severe deafness (despite hearing aid). The token test is administered before the recruitment in case of doubt.
- Verbal expression ability below 75% in an ordinary conversation, even with facilitation by the caregiver. A simple oral fluency test (verbal fluency by phonemic category) is administered before enrollment in case of doubt.
- Inability to participate in videoconferences due to unviable technical requirements (i.e., unavailability of computer, smartphone or tablet, webcam, microphone or speakers and failure to install Microsoft Teams and unavailability of a good Internet connection)
- Lack of support from a family member or friend if barriers due to technical knowledge or motor disability prevent the participant from participating in the video conferences despite having the necessary technical requirements.

### Informed consent

Participants must sign written consent forms for study participation and personal data handling and management.

### Assessment

The qualitative research at the basis of the interviews represents one of the gold standards to identify facilitators and barriers impacting the usability of AT systems [7]. If the knowledge surrounding a given phenomenon is still uncertain, or the researcher suspects the existence of hidden factors, interviews can undoubtedly provide support [37–39]. The data emerging from the verbatim transcription of the interview speech is analyzed by sorting the text into concepts, categories, and themes to be considered in the development of MAIA technology [38].

### Study procedure

The enrolment of eligible participants takes place with a meeting in person or within a teleconference. Within this meeting, the eligible participant is informed about project aims, design, and operational study modalities using a detailed information sheet, making explicit that interviews will be recorded and stored to allow analysis. During the meeting, the potential participant is stimulated to ask questions about the study. Then, should the subject want to participate, the enrollment is formalized by acquiring informed consent upon successfully verifying the inclusion and exclusion criteria. Next, the participant will sign the consent form physically in case of meeting in person or electronically in a teleconference. Upon verification of the participant’s identification document, the researcher will read the consent form, asking to state explicitly whether the subject accepts or refuses to participate in the study. Then, the researcher will document in the consent form the willingness of the subject to participate. The procedure will be video recorded and digitally stored on a secure server.

Once the consent is provided, the enrolling researcher pseudonymizes the participant to secure their anonymity. Following this, primary clinical data (i.e., a brief history of the injury and disability profile) is collected into a Case Report Form. Then each participant is randomized into any of the two available interview modalities, i.e., individual interview or focus group. There are two separate blocked randomization lists (block size: four), one for patients and one for caregivers. Following randomization, the interviewers contact the enrolled participant to make an appointment for the actual interview. Focus groups may be heterogeneous by aetiology (especially in case of difficulties in participants recruitment). However, the respondent’s focus group is homogenous, so that patients and caregivers are segregated into different focus groups. Each group is expected to consist of four participants.

Interviews with single participants and focus groups are similar in structure and conducted by an experienced psychologist in qualitative research, who follow the procedure described in the S1 Appendix. The interview is composed of open questions prompting participants’ narrative flows, and the individual consultation setting facilitates the collection of information about a specific topic. On the other hand, the focus group allows discussion between participants so that new information may be generated by mutual and social exchange [40–42]. For each DTCP group, a couple of individual interviews are carried out as pivotal. Interview analysis prompts project researchers to adjust their structure according to specific needs.

Each interview or focus group begin with the psychologist/moderator introducing themselves and inviting participants to do the same. Then, the interviewer describes the discussion’s objectives: developing a user-friendly AI system for controlling neuroprosthesis and other assistive devices capable of improving the quality of patients’ daily lives. Finally, the MAIA project and the human-centred AI is introduced using a PowerPoint presentation (available at https://site.unibo.it/maia-fetproact/en/per-pazienti-e-caregiver) embedding an explanatory video (https://youtu.be/AJRfGlstEic).

Following this, the participants are invited to report their previous experiences with AT, such as electronic wheelchairs, exoskeletons, prosthetic arms, or legs. They are also asked to express their opinions about the project, focusing on strengths and weaknesses. Thus, the interviewer invites participants to envision what system features might improve their autonomy in daily life activities (i.e., usefulness, control, manipulation, ease of use, others) and describe them. After that, the psychologist/moderator drive participants to express their support or opposition about possible future use of the system, even highlighting that, as it appears in the videos of presentation, the control of the technological systems as MAIA might be not that straight, and require some training. Subsequent topics that the interviewer brings about regard neuronal implants and related fears of mind control (i.e., thoughts to ill-interpreted or personality to be changed). Then, the discussion moves on to the possible control channels for the neuroprosthesis/devices (i.e., visual, visual-acoustic, visuotactile, other). Finally, participants are asked to express their availability to test the prototype (i.e., the AI system and neuroprosthesis) and raise any other issues that emerged until that moment (See S1 Appendix for Interview and focus group structures).

### Sample size and statistical analysis

Recruitment stops once saturation is reached. Saturation occurs when no new categories (see below) emerge for three consecutive interviews [38,39]. Previous works suggest that the content saturation might be reached with 30 participants, 15 PWD, and 15 caregivers for each DTCP line [34,41–43].

### Data analysis

Interview content analysis is carried out over the texts extracted from the recorded videos and reported verbatim. The analysis regards the yielding of patterns of meaning within the data [44]. It follows the deductive analysis of the texts via keyness-driven annotation of codes and their clustering in conceptual themes [39,42,44–46].

Data gathered from participants (either from interviews or focus groups) is pooled and analyzed together: as we expect similar needs within the same DTCP but different needs across DTCP, data from different DTCP are kept distinct and analyzed separately.

Socio-demographic, clinical, and psycho-social variables, such as gender, age, level of fragility, time from injury, are the factors that, together with intrinsic motivation and social pressure, are expected to modulate interviews outcomes. Notwithstanding the hypotheses, in the case of additional elements, they are analyzed and considered in the final thematic map.

### Ethics and dissemination

The project protocol was approved by the Local Ethics Committees (ASL_BO n. 0031849 provided on 29/03/2021; UNIBO n. 284787 provided on 05/11/2021) before commencing the actual recruitment. The study is performed according to the principle of the Helsinki Declaration. The study results are disseminated in peer-reviewed scientific journals and in abstract format at scientific events. Once the data collection is terminated, they will be available from the following URL http://amsacta.unibo.it/id/eprint/6854.

### Status and timeline of the study

Participants’ recruitment has commenced on April 2021 and has not been completed yet. A preliminary analysis will be conducted in 2022, and the final report is expected to occur in late 2022.

## Results and discussion

Despite the data on disability and the progress of knowledge related to neurogenesis and neuronal plasticity, a complete recovery based on the regeneration of damaged nerve tissue cannot be offered. Alternative to tissue regeneration, the technologies based upon brain-human interfaces and AI appear to support intelligent neuroprostheses or robotic devices developed to assist the damaged neuronal networks. In this light, the current project gathers a series of academic and commercial partners to build an AI software interface to guide the sensorimotor control of robotic devices for people with severe motor disabilities. User-centered design principles are adopted to accomplish the task. The end-users enrolled in this project include persons with severe disabilities due to stroke, SCI, or severe brain injuries and their caregivers. They are interviewed about their experience with previous AT, the problems and the solutions they envision the system can present, and their intention to be enrolled in a prototype implementation program.

The expected results span several areas. The first area may regard the technical aspects of the device, health-related components, setting components, while the second one may represent psycho-social issues concerning with technology acceptance model [47]. In particular, the latter problems are expected to revolve around intrinsic motivational and extrinsic social aspects, aspects concerning the usefulness of the MAIA system, and the related ease to use. Moreover, the last area may involve individual factors such as age, gender, the levels of fragility, psychological aspects involved in the mental representation of the body image, personal endurance, and tolerance toward AT-related burdens. In particular, the use of AT might be associated with a detrimental perception of stigma, which may generate negative expectations or even refrain either PWD or caregivers from envisioning optimistic scenarios about living with such prosthesis [48]. Older caregivers, indeed, tend to embrace more negative attitudes towards disability itself than their younger counterparts, as time affects the disability burden [49]. On the other hand, the longer the time from the disability onset, the higher the probability that PWDs develop positive attitudes towards their disability [49] due to the embodiment of the neuroprosthesis in their daily living [24].

To conclude, the end-user-driven acknowledgement of the features that might humper or foster the end-user acceptability of a human-centered AI system like MAIA would shorten system development while facilitating end-user adoption.

### Protocol implementation

The protocol described in the paper capitalises on the EU HORIZON 2020 Program, whose proposal fell within cutting-edge high-risk / high-reward research and innovation projects. The present protocol considers gender, fragility levels, psychological aspects involved in the mental representation of body image, personal endurance, and tolerance toward AT-related burdens. However, if cultural effects emerge, authors will consider implementing a cross-nation design.

## Supporting information

S1 Appendix (DOCX)

## Abbreviations

TBI: Traumatic Brain Injury
SCI: Spinal Cord Injury
PWD: People With Disability
AT: Assistive technologies
AI: Artificial Intelligence
PPC: Posterior Parietal Cortex
DTCPs: Diagnostic–Therapeutic Care Pathways
Stroke: Brain Stroke
CRF: Case Report Form
IRCCS: Istituto di Ricovero e Cura a Carattere Scientifico (Scientific Institute for Hospitalization and Treatment)
GCS: Glasgow Come Scale
