# Supplementary material for: A Multifunctional Adaptive and Interactive AI system to support people living with stroke, acquired brain or spinal cord injuries: A study protocol
Source: PLoS One. 2022 Apr 11;17(4):e0266702. doi: 10.1371/journal.pone.0266702 (PMC9000091; doi:10.1371/journal.pone.0266702)
Supplement: S1 Appendix — (DOCX) [file pone.0266702.s001.docx]

## **Appendix**

### Individual interview / Focus group

Introduction:

Thank you for participating in our research on the MAIA project.

We are interested in developing an Artificial Intelligent system to improve people's autonomy with acquired motor disabilities due to injuries to the neural system.

We are interested in listening to your opinions and suggestions about the MAIA system to develop a user-friendly system that could improve the quality of your daily life.

We record the interview, so do not miss any information, and we guarantee your privacy. The discussion lasts around 90 minutes. There are no wrong or correct answers.

1. Could you kindly introduce yourself with your name and where you live, please?
2. May I ask you why you decided to participate in the interview?

We are going to present to you the project: (Showing MAIA PowerPoint file)

1. Have you ever had (or do you have right now) experiences with any technological devices that support you in your daily activities?

YES: a) Could you kindly describe the technology you are using or have used, please?.

b) How is/was your experience with this technology? Could you describe any positive or negative issues?

NO: a) Why have you never used any technological devices?

b) Would you be willing to use them in the future?

1. What do you think about the MAIA project?
2. What could be the strong and weak points of the system?
3. What features should technology have to improve people's autonomy in daily life activities (e.g., usefulness, control, manipulation, ease of use, etc.)? Would you describe any of its features, please?
4. Would you use this technological system?
5. Can you envision any reasons not to use the system?
6. Have you any worries or contrarieties about the system?
7. Now, let's turn back to the MAIA project: the user of the system may control it through a sensor implanted in the brain:
8. What is your opinion about this? In particular:
   1. Do you think that MAIA may control you (e.g., your movements, your thoughts, etc.)?
   2. Do you think that the sensor may change your personality?
9. Would you trust a technological system with partial control over your activities?
10. Imagine that the system needs to ascertain if it has correctly interpreted your (motor) intention, such as drinking a glass of water. How would you prefer the system to confirm it? Here we have some options: through a screen (visual), vibrotactile feedback (touch), sound feedback (hear). Or would you fancy some other strategy? What kind of channel would you like to use? Would you like to use eye movements, or do you not trust them?
11. Would you be available to test the system when the proof of concept is ready?

We have finished with the questions; would you add any other issues/suggestions?

Thanks for your time, and please do not hesitate to contact us if you have any further comments.
